# Influence of Different Hip Joint Centre Locations on Hip and Knee Joint Kinetics and Kinematics During the Squat

**DOI:** 10.2478/hukin-2014-0106

**Published:** 2014-12-30

**Authors:** Jonathan Sinclair, Stephen Atkins, Hayley Vincent

**Affiliations:** 1 Division of sport Exercise and Nutritional Sciences, School of Sport Tourism and Outdoors, University of Central Lancashire.

**Keywords:** hip joint centre, biomechanics, kinematics

## Abstract

Identification of the hip joint centre (HJC) is important in the biomechanical examination of human movement. However, there is yet to be any published information regarding the influence of different HJC locations on hip and knee joint kinetics during functional tasks. This study aimed to examine the influence of four different HJC techniques on 3-D hip and knee joint kinetics/kinematics during the squat. Hip and knee joint kinetics/kinematics of the squat were obtained from fifteen male participants using an eight camera motion capture system. The 3-D kinetics/kinematics of the squat were quantified using four hip joint centre estimation techniques. Repeated measures ANOVAs were used to compare the discrete parameters as a function of each HJC location. The results show that significant differences in joint angles and moment parameters were evident at both the hip and knee joint in the coronal and transverse planes. These observations indicate that when calculating non-sagittal joint kinetics/kinematics during the squat, researchers should carefully consider their HJC method as it may significantly affect the interpretation of their data.

## Introduction

Accurate identification of the hip joint centre (HJC) is essential in the biomechanical examination of human movement as it allows the mechanics of the hip and knee joints to be studied during sport and clinical tasks (Camomilla et al., 2006; Piazza et al., 2004; Sangeux et al., 2011).

There are a number of techniques currently available in biomechanical literature for the identification of the HJC. These include marker based techniques whereby the position of the HJC is estimated relative to anatomic pelvic landmarks (Bell et al., 1989; Seidel et al., 1995; Schofer et al., 2010). The first marked based method was initiated by Bell et al. (1989) in which the HJC was positioned relative to the anterior super iliac spine markers. In addition further anatomical methods are available which determine the HJC relative the greater trochanter landmark. Two methods are available using this approach; the first developed by Weinhandl and O’Connor (2010) places the HJC at one-quarter of the distance from the ipsolateral to the contralateral greater trochanter. The second approach estimates the HJC using a predetermined medial projection of 0.089 m from the greater trochanter (Sinclair et al., 2012). Finally more recent work has advocated the adaptation of functional techniques for the identification of the HJC. Functional techniques estimate the centre of rotation of the thigh segment relative to the pelvis using 3-D kinematic data (Leardini et al., 1999; Marin et al., 2003; Piazza et al., 2001; Shea et al., 1997).

Each of the aforementioned techniques has been utilized in biomechanical literature to quantify the position of the HJC. However, little consideration is usually given to how variations in HJC location may influence the resultant joint mechanics at the hip and knee. Previous analyses have examined the differences in 3-D kinematic parameters as a function of different HJC locations. Sinclair et al. (2012) compared 3-D hip joint kinematics during running using two different HJC locations. It was shown that sagittal plane kinematics differed significantly between the two methods. Furthermore, Sinclair et al. (2013) investigated the influence of three different HJC techniques on hip and knee joint kinematics during the fencing lunge. They showed that whilst the kinematic waveforms were similar, significant differences in discrete parameters were also evident at both the hip and knee joint in the coronal and transverse planes.

However, it should be noted that whilst these investigations provided information regarding the effects of different HJC locations on measures of joint angulation during running and fencing activities, there is yet to be any published information regarding the influence of different HJC locations on hip and knee joint kinetics during functional tasks such as the squat. Therefore, the aim of the current investigation was to examine the influence of four different HJC techniques on 3-D hip and knee joint kinetics/kinematics during the squat. A study of this nature may provide information to biomechanists regarding the influence of difference HJC algorithms on clinically relevant outcome measures at the hip and knee joints.

## Material and Methods

### Participants

Fifteen male participants volunteered to take part in this investigation. All were experienced in squat lifting and free from musculoskeletal pathology at the time of data collection. Participants all provided written informed consent in accordance with the Declaration of Helsinki. The mean characteristics of the participants were: age 26.54 ± 6.21 years, body height 1.80 ± 0.10 m, body mass 79.47 ± 6.88 kg, BMI 24.20 ± 1.49 and body fat % 12.48 ± 2.99. Ethical approval for this project was obtained from the University of Central Lancashire School of Sport Tourism and Outdoors ethical committee.

### Procedure

Participants completed five back squat repetitions using their normal squat technique. Participants lifted 70% of their 1 repetition maximum, which was selected on the basis of the recommendations provided by Brzycki (1993). To acquire joint kinetic information the right foot was positioned onto a piezoelectric force platform (Kistler, Kistler Instruments Ltd., Alton, Hampshire).

Kinematic information was captured at 250 Hz using an eight camera optoelectric motion analysis system (Qualisys^TM^ Medical AB, Goteburg, Sweden). This investigation utilized the calibrated anatomical systems technique (CAST) (Cappozzo et al., 1995). In order to define the pelvis, right thigh and shank segments, retroreflective markers (19 mm) were positioned on the medial and lateral malleoli, medial and lateral epicondyle of the femur, greater trochanter of the right leg, anterior superior iliac spines (ASIS) and posterior superior iliac spines (PSIS). Technical tracking clusters comprised of four markers mounted to a thin sheath of lightweight carbon fiber, were positioned on the shank, thigh and pelvis. For all segments, the positive Z (transverse plane) axis was defined in the direction of distal to proximal joint centres. The positive Y (coronal plane) axis was defined as perpendicular to the Z axis and while the X (sagittal) axis was delineated as a cross-product of Y and Z axes.

The ‘Bell’ technique was based on previously outlined recommendations (Bell et al., 1989) using the width of the ASIS markers. This procedure positioned the HJC 14% of the ASIS breadth medially, 19% posteriorly, and 30% distally from the ipsilateral (Right) ASIS. The first projection method ‘1/4 GT width’ was also based on previously established guidelines (Weinhandl and O’Connor, 2010), this method estimates the HJC as a three-dimensional point, located at one-quarter of the distance along a line from the ipsolateral (Right) to the contralateral (Left) greater trochanter markers. The second projection method ‘0.089 m’ positions the HJC using a medial projection of 0.089 m from the greater trochanter marker (Sinclair et al., 2012).

The ‘functional’ method used the motion based techniques outlined by Schwartz and Rozumalski (2005). This calculation used rotations between the pelvis and thigh segments to calculate an instantaneous axis of rotation. The average intersection point of these segments provides the location of the HJC. To define the functional HJC, the participants performed five sequential flexion-extension and abduction-adduction movements of the right hip at a self-selected velocity followed by a cycle of full hip circumduction. Flexion-extension and abduction-adduction ranges of movement were in the order of 45 and 40° in accordance with Leardini et al. (1999).

### Data processing

Trials were digitized using Qualisys Track Manager to identify anatomical and cluster markers. Files were then exported in C3D format and kinetic/kinematic parameters were quantified using Visual 3-D (C-Motion, Germantown, MD, USA) after the data had been smoothed using a Butterworth low pass 4th order zero-lag filter at a cut off frequency of 6 Hz. Hip and knee joint kinematics were quantified using an XYZ sequence of rotations. All kinetic/kinematic waveforms were time normalized to 100% of the squat movement. Kinematic measures from the hip and knee extracted for statistical analysis were 1) peak angle during the squat movement and 2) relative range of motion (ROM) from initiation of movement to the peak angle. Kinetic parameters from the aforementioned joints were 1) peak joint moment, 2) peak joint force, 3) peak positive work, 4) peak negative work.

A predictive algorithm was utilized to quantify patellofemoral contact force (PTCF) and patellofemoral contact pressure (PTCP) (Ward and Powers, 2004). PTCF (B.W) were estimated using knee flexion angle (KFL) and knee extensor moment (KM) through the biomechanical model of Ho et al. (2012). The moment arm of the quadriceps (QMA) was calculated as a function of KFA using a non-linear equation, based on cadaveric information presented by Eijden et al. (1986):

$$\text{QMA}=0.00008\;{\text{KFL}}^{3}-0.013\;{\text{KFA}}^{2}+0.28\;\text{KFL}+0.046$$

Quadriceps force (FQ) was calculated using the below formula:

FQ=KM/QMA

PTCF was estimated using the FQ and a constant (C):

PTCF=FQ C

The C was described in relation to KFL using the equation described by van Eijden et al. (2012):

$$C=(0.462+0.00147\;{\mathrm{KFL}}^{2}-0.0000384\;{\mathrm{KFL}}^{2})/(1-0.0162\;\mathrm{KFL}+0.000155\;{\mathrm{KFL}}^{2}-0.000000698\;{\mathrm{KFL}}^{3})$$

PTCP (MPa) was calculated using the PTCF divided by the patellofemoral contact area. The contact area was delineated by fitting a 2nd-order polynomial curve to the data of Salsich et al. (2003) showing patellofemoral contact areas at varying levels of KFL.

PTCP=PTCF/contact area

### Statistical analyses

Means and standard deviations from the 3-D kinetic and kinematic parameters were calculated for each hip joint centre location technique. Differences between the parameters were examined using one-way repeated measures ANOVA with significance accepted at the p ≤ 0.05 level (Sinclair et al., 2013). Post-hoc pairwise comparisons were conducted on all significant main effects using a Bonferroni adjustment to control type I error. Effect sizes were calculated using an eta^2^ (η^2^). If the sphericity assumption was violated then the degrees of freedom were adjusted using the Greenhouse-Geisser correction. All statistical procedures were conducted using SPSS 21.0 (IBM, SPSS Inc., USA).

## Results

### Joint angles

A significant main effect (F_(3, 42)_ = 9.87, p≤0.05, η^2^=0.56) was observed for the magnitude of peak hip abduction. Post-hoc analysis revealed that peak hip abduction was significantly (p<0.05) greater in the functional condition compared to the other HJC configurations. In addition a significant main effect (F_(3, 42)_ = 10.57, p≤0.05, η^2^=0.59) was also shown for the magnitude knee relative ROM in the coronal plane. Post-hoc analysis revealed that a relative ROM was significantly (p<0.05) greater in the functional condition compared to the other HJC configurations. Finally, a significant main effect (F_(3, 42)_ = 10.24, p≤0.05, η^2^=0.57) was also shown for the magnitude peak knee internal rotation. Post-hoc analysis revealed that peak knee internal rotation was significantly (p<0.05) greater in the functional condition compared to the other HJC configurations.

### Joint moments

A significant main effect (F (3, 42) = 12.54, p≤0.05, η2=0.63) was observed for the magnitude of the peak hip adduction moment. Post-hoc analysis revealed that the peak hip adduction moment was significantly (p<0.05) greater in the functional condition compared to the other HJC configurations. In addition, a significant main effect (F (3, 42) = 12.09, p≤0.05, η2=0.61) was also shown for the magnitude of the peak knee external moment. Post-hoc analysis revealed that the peak external moment was significantly (p<0.05) greater in the functional condition compared to the other HJC configurations.

### Joint forces

A significant main effect (F (3, 42) = 8.57, p≤0.05, η2=0.49) was observed for the magnitude of peak knee medio-lateral force. Post-hoc analysis revealed that peak medial force was significantly (p<0.05) lower in the functional condition compared to the other HJC configurations.

### Joint powers

No significant (p>0.05) differences in joint powers were observed as a function of the four different HJC configurations.

### Patellofemoral kinetics

No significant (p>0.05) differences in patellofemoral joint kinetics were observed as a function of the four different HJC configurations.

## Discussion

The current investigation aimed to examine the influence of four different HJC techniques on 3-D hip and knee joint kinetics/kinematics during the squat movement. This study represents the first investigation to consider the influence of these four HJC configurations on both joint kinetic and kinematic parameters.

With regard to the discrete parameters in the sagittal plane, no significant differences were observed for the hip or knee joint between any of the four HJC configurations. However, in the coronal and transverse planes, significant differences were mediated as a function of the four HJC location techniques. These observations concur with those of Sinclair et al. (2013) who showed that non-sagittal parameters were significantly influenced as a function of different HJC location methods. It should be noted that the functional HJC configuration was shown to be habitually different from the other methods used in this study. It was hypothesized that this was due to the more medial position of the HJC with the functional method, in comparison to the other configurations. As the segment axes were defined as a function of both the proximal and distal end points the position of the thigh segment was influenced by the medial positioning of the functional HJC. This led to the thigh segment being more adducted and externally rotated and had the additional effect of altering the magnitude of the coronal and transverse plane moments on the hip and knee joints.

The findings from the current investigation may have potential relevance to the clinical interpretations of joint kinetic/kinematic information. Excessive coronal and transverse plane rotations/moments of the hip and knee joint, which were shown to be significantly greater when the functional HJC was used, have been linked to the aetiology of degenerative pathologies in dynamic movements (Mizuno et al., 2001; Horton and Hall, 1989). Therefore, it appears based on these findings that when calculating non-sagittal joint kinetics/kinematics during the squat, researchers should carefully consider their HJC method as it may significantly affect the interpretation of their data.

It is important to state, however, that the different HJC configurations had little influence on the measures of patellofemoral kinetics and hip/knee joint powers. This leads to the conclusion that whilst variations in HJC location can significantly influence joint angles/moments, there does not appear to be any effects when quantifying patellofemoral kinetics or hip/knee joint powers. It appears therefore that the selection of HJC location is perhaps less of a concern when quantifying joint powers and patellofemoral loads.

There are some limitations to the current investigation that are important to be acknowledged so that future analyses may address them. The current investigation utilized an all-male sample what may serve as a potential limitation to the current study. Females are associated with different pelvic dimensions and body fat percentage in comparison to age matched males (Morris et al., 1982), which may make anatomical marker placement more difficult and influence the effectiveness of projected methods of establishing the HJC. Therefore, it is recommended that the current investigation be repeated using a female sample. The fact that radiographic measurements were not obtained as part of the current work may also be a potential limitation as the accuracy of each method in terms of its ability to locate the true anatomical position of the HJC was not determined. Although radiographic measurements represent a somewhat invasive technique that requires more encompassing ethical authorization, future work should seek to address this factor in both males and female subjects before an optimal HJC estimation technique can be recommended.

In conclusion, the current investigation adds to current knowledge by providing a comprehensive examination of the effects of different HJC configurations on both kinetic and kinematic parameters. Whilst it is beyond the scope of the information generated in the current study to determine an optimal technique for the quantification of the HJC, it provides important information as different HJC techniques mediated significantly different kinetic/kinematic parameters when in the coronal and transverse planes. It can be determined based on these observations that different HJC location procedures should not be used interchangeably and cross-study comparisons of hip and knee joint kinetics/kinematics outside of the sagittal plane should be made with caution if different HJC locations have been used.

## Figures and Tables

**Figure 1:**
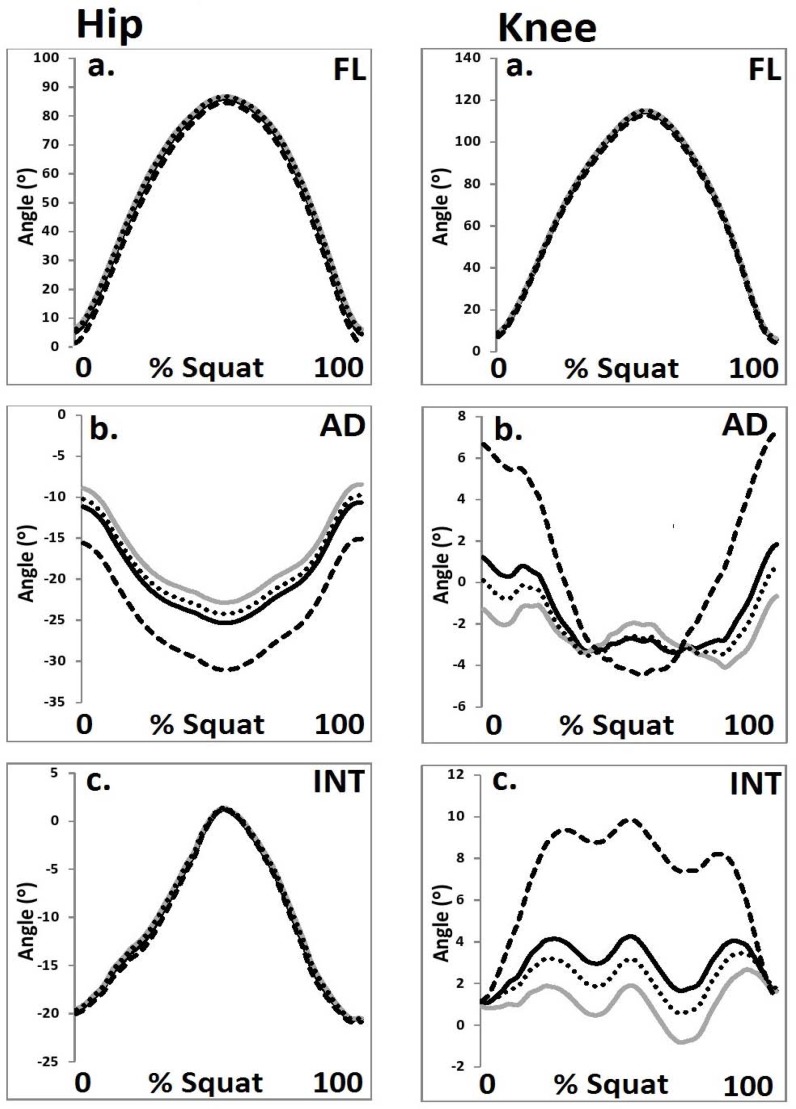
Hip and knee joint angles from each HJC configuration in the a. sagittal, b. coronal and c. transverse planes (black = Bell, grey = 0.089 m, dot = ¼ GT width, dash = functional) (FL = flexion, AD = adduction, INT = internal).

**Figure 2:**
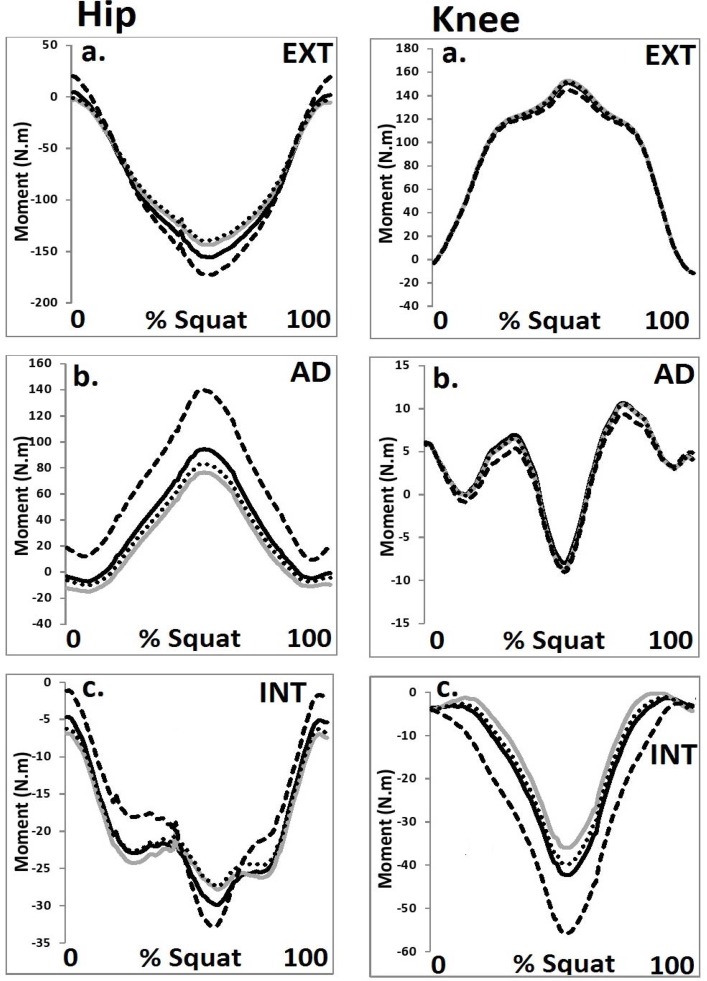
Hip and knee joint moments from each HJC configuration in the a. sagittal, b. coronal and c. transverse planes (black = Bell, grey = 0.089 m, dot = ¼ GT width, dash = functional) (EXT = external, AD = adduction, INT = internal).

**Figure 3:**
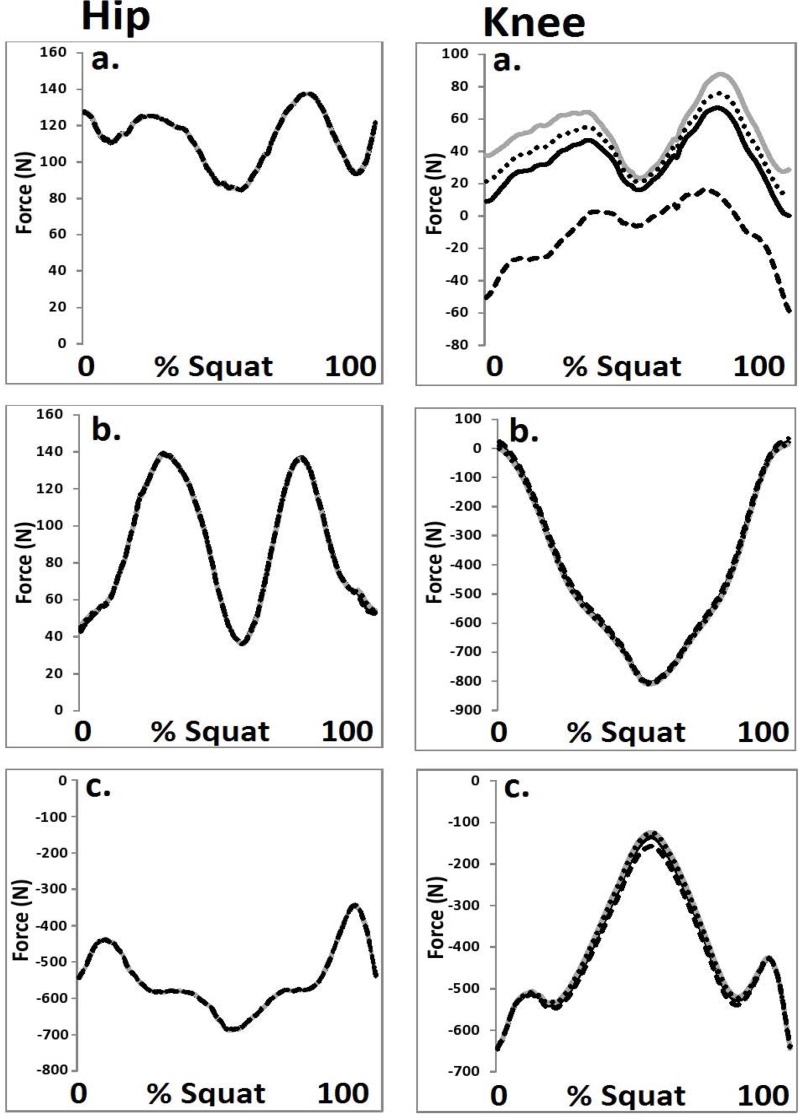
Hip and knee joint forces from each HJC configuration in the a. medio-lateral, b. anterior-posterior and c. vertical directions (black = Bell, grey = 0.089 m, dot = ¼ GT width, dash = functional).

**Figure 4:**
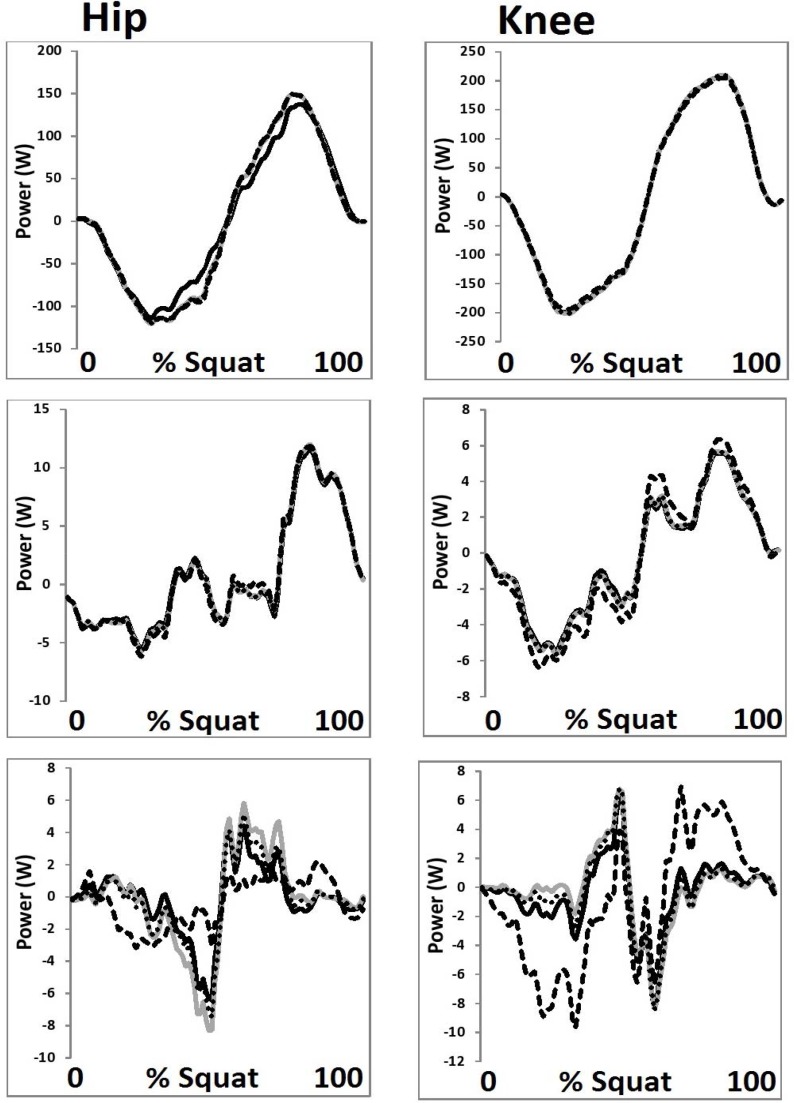
Hip and knee joint powers from each HJC configuration in the a. sagittal, b. coronal and c. transverse planes (black = Bell, grey = 0.089 m, dot = ¼ GT width, dash = functional).

**Figure 5:**
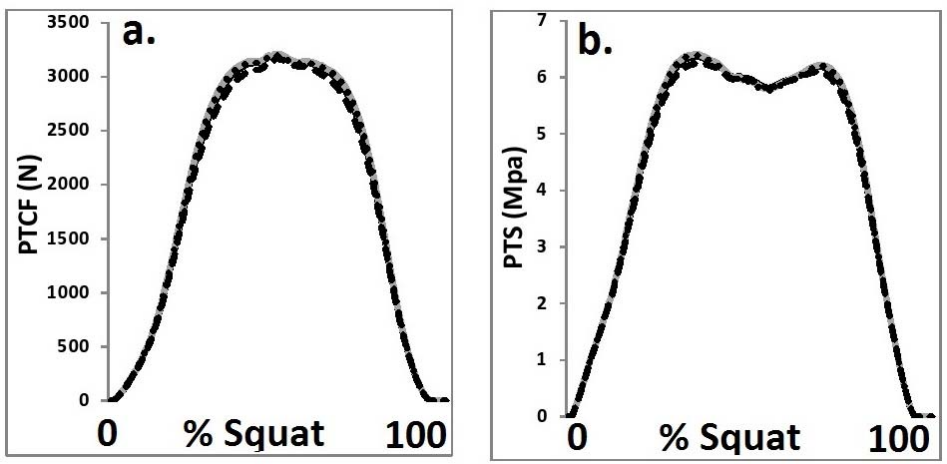
Patellofemoral kinetics from each HJC configuration a. patellofemoral contact force, b. patellofemoral contact pressure (black = Bell, grey = 0.089 m, dot = ¼ GT width, dash = functional).

**Table 1:** Hip and knee joint angles as a function of each HJC configuration.

	**Bell**		**Projected (0.089 m)**		**Projected (1/4 GT width)**		**Functional**	

	**Mean**	**SD**	**Mean**	**SD**	**Mean**	**SD**	**Mean**	**SD**
	
**Hip**								
**Sagittal plane (+ = flexion/ − = extension)**								
Peak flexion (°)	86.39	12.54	86.14	12.87	86.09	13.05	84.30	12.80
Relative ROM (°)	82.34	4.23	83.01	4.98	82.89	4.11	82.61	4.03
**Coronal plane (+ = adduction/ − = abduction)**								
Peak abduction (°)	−23.61	5.21	−21.42	4.65	−22.65	4.60	−33.59	5.13
Relative ROM (°)	12.79	2.54	12.67	3.06	12.81	2.61	16.04	2.46
**Transverse plane (+ = internal/ − = external)**								
Peak internal rotation (°)	0.56	3.21	0.68	3.51	0.52	3.47	0.63	3.09
Relative ROM (°)	20.09	2.99	20.81	3.17	20.73	3.20	20.44	3.08
**Knee**								
**Sagittal plane (+ = flexion/ − = extension)**								
Peak flexion (°)	115.37	15.22	115.89	14.61	115.63	14.75	112.69	15.08
Relative ROM (°)	104.87	5.68	104.06	5.43	105.12	5.11	104.20	5.26
**Coronal plane (+ = adduction/ − = abduction)**								
Peak abduction (°)	−3.68	3.21	−3.40	3.46	−3.56	3.28	−4.59	3.03
Relative ROM (°)	4.31	2.11	1.56	2.54	3.66	2.38	11.31	2.14
**Transverse plane (+ = internal/ − = external)**								
Peak internal rotation (°)	3.89	2.55	1.43	2.31	3.06	2.20	9.37	2.56
Relative ROM (°)	2.86	1.05	0.48	1.11	1.06	1.49	8.81	1.30

**Table 2:** Hip and knee joint moments as a function of each HJC configuration.

	**Bell**		**Projected (0.089 m)**		**Projected (1/4 GT width)**		**Functional**	

	**Mean**	**SD**	**Mean**	**SD**	**Mean**	**SD**	**Mean**	**SD**
	
**Hip**								
**Sagittal plane**								
Peak moment (N·m ·kg)	−158.41	32.54	−141.63	31.28	−139.59	29.65	−176.84	32.97
**Coronal plane**								
Peak moment (N·m ·kg)	89.54	22.87	69.38	23.62	77.51	22.43	138.63	22.26
**Transverse plane**								
Peak moment (N·m ·kg)	−29.82	13.57	−26.26	14.25	−26.10	13.86	−33.40	13.63
**Knee**								
**Sagittal plane**								
Peak moment (N·m ·kg)	146.68	30.21	147.09	29.64	146.13	29.31	140.20	31.06
**Coronal plane**								
Peak moment (N·m ·kg)	−7.46	2.54	−8.01	2.88	−8.13	2.74	−8.15	2.39
**Transverse plane**								
Peak moment (N·m ·kg)	−42.51	8.64	−34.21	8.12	−38.59	8.06	−56.40	7.94

**Table 3:** Hip and knee joint forces as a function of each HJC configuration

	**Bell**		**Projected (0.089 m)**		**Projected (1/4 GT width)**		**Functional**	

	**Mean**	**SD**	**Mean**	**SD**	**Mean**	**SD**	**Mean**	**SD**
	
**Hip**								
**Medio-lateral**								
Peak force (N)	136.71	23.54	135.89	25.47	136.07	23.09	133.98	24.15
**Anterior-posterior**								
Peak force (N)	140.20	26.87	139.68	26.42	140.07	26.08	140.17	26.35
**Vertical**								
Peak force (N)	−650.51	84.21	−649.38	85.63	−651.28	85.17	−651.32	84.86
**Knee**								
**Medio-lateral**								
Peak force (N)	52.19	10.22	85.39	9.89	67.85	10.17	4.88	10.10
**Anterior-posterior**								
Peak force (N)	−810.09	100.57	−811.29	98.63	−812.81	100.08	−812.60	99.22
**Vertical**								
Peak force (N)	−153.19	27.54	−147.80	26.85	−145.24	27.13	−170.53	26.97

**Table 4:** Hip and knee joint powers as a function of each HJC configuration.

	**Bell**		**Projected (0.089 m)**		**Projected (1/4 GT width)**		**Functional**	

	**Mean**	**SD**	**Mean**	**SD**	**Mean**	**SD**	**Mean**	**SD**
	
**Hip**								
**Sagittal plane**								
Peak positive work (W)	136.81	21.54	147.21	22.77	146.69	21.83	147.88	22.13
Peak negative work (W)	−120.18	19.51	−126.61	20.29	−127.54	19.87	−127.43	20.14
**Coronal plane**								
Peak positive work (W)	11.37	2.58	12.59	2.61	13.00	2.43	12.83	2.67
Peak negative work (W)	−7.61	2.14	−8.05	2.26	−7.37	2.13	−7.80	2.07
**Transverse plane**								
Peak positive work (W)	4.21	2.03	6.03	2.11	5.65	2.15	2.43	2.09
Peak negative work (W)	−4.47	2.15	−7.64	2.09	−7.17	2.17	−2.63	2.16
**Knee**								
**Sagittal plane**								
Peak positive work (W)	207.09	33.22	210.34	32.09	208.61	32.43	203.04	32.00
Peak negative work (W)	−192.84	27.61	−191.71	26.38	−193.63	26.85	−188.14	26.88
**Coronal plane**								
Peak positive work (W)	5.36	1.55	5.09	1.67	5.43	1.51	6.46	1.49
Peak negative work (W)	−5.15	1.44	−5.22	1.48	−5.01	1.57	−6.23	1.53
**Transverse plane**								
Peak positive work (W)	6.67	2.38	6.50	2.74	6.63	2.29	6.95	2.67
Peak negative work (W)	−7.38	2.87	−7.24	2.93	−7.66	2.84	−9.12	2.79

**Table 5:** Patellofemoral kinetics as a function of each HJC configuration.

	**Bell**		**Projected (0.089 m)**		**Projected (1/4 GT width)**		**Functional**	

	**Mean**	**SD**	**Mean**	**SD**	**Mean**	**SD**	**Mean**	**SD**
	
**Peak PTCF (N)**	3245.55	325.22	3274.11	318.65	3258.49	322.88	3179.12	305.63
**Peak PTS (Mpa)**	6.34	1.87	6.41	1.94	6.38	1.85	6.19	1.78
